# Nurses’ competencies in home healthcare: an interview study

**DOI:** 10.1186/s12912-017-0264-9

**Published:** 2017-11-17

**Authors:** Henrik Andersson, Maria Lindholm, Margareta Pettersson, Lise-Lotte Jonasson

**Affiliations:** 1 University of Borås, Faculty of Caring Science, Work Life and Social Welfare, Borås, Sweden; 2 Centre for Adult Education, Härryda municipality, SE-435 80 Mölnlycke, Sweden

**Keywords:** Home healthcare, Nurses, Competencies, Competence development, Sustainable development, Content analysis

## Abstract

**Background:**

Nurses working in Home healthcare (HHC) are facing major challenges since more advanced care and treatment are increasingly being carried out in patients’ homes. The aim of this study has been to explore how nurses experience their competencies in HHC situations.

**Methods:**

This study has a qualitative and explorative design. Ten nurses were interviewed and data was analyzed using content analysis.

**Results:**

The themes “Being a capable nurse”, “Being a useful nurse” and “Being a subordinate and dependent nurse” were identified. Nurses want to be capable of taking care of patients, to develop their competencies and to perform their duties in the way required. They also want their work to be useful and to provide good and safe HHC. Finally, nurses want to improve HHC care by applying their competencies. Simultaneously, they are subordinate and dependent in relation to their manager and also dependent upon their manager’s interest in encouraging nurses’ competence development.

**Conclusions:**

Nurses in HHC are responsible for many seriously ill patients and they want to contribute to good and safe patient care. To maintain patient safety, reduce the risk for burnout and staff turnover as well as to contribute to a sustainable development of the work, strategies for transferring competencies between nurses and efforts for competence development are needed.

## Background

Nurses working in Home healthcare (HHC) are facing major challenges since more advanced care and treatment are increasingly being carried out in patients’ homes. In this study, patients’ homes are defined as residential facilities or ordinary housing. Nurses in HHC provide healthcare for patients who are terminally or chronically ill, recovering or disabled [1]. Examples are seriously ill patients whose care requires different kinds of technical apparatus e.g. central line, port-a-cath, pain pump or peritoneal dialysis [2] and patients receiving palliative care who wishes to die at home [3]. In this study, HHC refers to different interventions i.e. medical treatment or nursing care, provided with or without delegation in patients’ homes or the equivalent by qualified nurses, both registered nurses (RNs) and assistant nurses (ANs) [4]. The term “nurses” is used for both RNs and ANs in this paper.

HHC could be seen from an international perspective. Simultaneously, Western countries, for example USA, Canada, Germany, Norway and Sweden have different healthcare systems that influence the care provided. Research has shown that staffing, staffing levels, standards and qualifications are conditions that are difficult to compare between countries [5]. Hence, this study offers a Swedish perspective regarding nurses’ competencies in HHC. The healthcare system in Sweden consists of 290 municipalities that are self-governing local authorities [6]. A municipality has the main responsibility for providing healthcare, support and service in patients’ homes [7]. The work in HHC is mostly carried out by RNs and ANs i.e. personnel who have formal education. However, there are some differences between RNs and ANs related to their training, roles and responsibilities in the care provided. In Sweden, RNs’ education includes 3 years of university studies resulting in a Bachelor of Science Degree in Nursing [8]. ANs have 3 years of upper secondary school studies that lead to a certification in healthcare [9]. RNs are expected to take an advisory role in HHC, which includes responsibility to supervise, guide, give instructions and delegate specific work-related issues to ANs, such as for example administering medicines. In contrast, ANs are expected to assist RNs in solving practical patient-related issues in HHC [10].

Nurses encounter patients with both medical and nursing needs that must be met in order for these patients to be able to live in their own homes. As a consequence, there is a wide range in nurses’ everyday activities, for example assessing health, giving treatment, conducting checkups and handling pharmaceutics [11], HHC nurses must also make decisions on certain issues [12, 13]. This means that nurses have significant responsibility for their patients’ care and treatment. However, care and treatment provided within HHC is different from the care given in hospital. The reason for this is that home is a place where the patients have their family and where e.g. certain values, preferences, culture and habits prevail [14]. These circumstances require health professionals to be capable of working independently and flexibly and to be able to provide advanced care and treatment based on patients’ individual healthcare needs in these patients’ homes.

Being prepared for open and flexible encounters with patients in complicated and complex situations is essential. Nurses are responsible for seriously ill patients, new treatments and medical equipment [15]. Assessments and decision-making are undertaken frequently in their everyday work, requiring advanced medical and nursing competencies [16]. Simultaneously, there are some challenges that have impact on nurses’ competencies. One challenge is the working environment. Research indicates that poor lighting, uncomfortable working positions, inadequate prescriptions and difficulties in maintaining sterility influence the care and treatment given to patients [3]. Another challenge is the workload. Studies show that heavy workloads make it difficult for nurses to update themselves on new knowledge and research, to improve the care given [15] and to deliver quality nursing care [17]. Research also indicates that nurses experience themselves as having great responsibility for their patients [18]. Simultaneously, studies show that nurses experience certain shortcomings in their competencies in areas such as assessment [19] and medical technology [3]. These shortcomings should be seen in the light of nurses having only limited opportunity to co-operate with and consult doctors and other colleagues for example in the evenings and at weekends [16, 20]. Hence, working without support at close hand requires nurses to have both medical and nursing competencies when encountering patients’ individual healthcare needs.

Competencies are an essential part in the practice of a profession [21]. However, this is a concept whose meaning can vary between different professions [22]. Knowledge is an important component of competence [21]. Knowledge can be described e.g. as knowing facts, being able to handle different situations or understanding the consequences of actions [23]. Competence includes both theoretical and practical knowledge. Which competencies are considered necessary in HHC is influenced by e.g. professional organizations, institutions of higher education and governmental agencies. Their judgement is based for example on ideas about which competencies are useful [24]. There are national competence descriptions that stipulate necessary competence for RNs in HHC [10, 12, 13], but not for ANs. Thus, there are no formal national requirements regarding competencies for all nurses in HHC.

In the light of the increased challenges posed by more advanced care and treatment of patients in their homes, the aim of this study has been to explore how nurses experience their competencies in HHC situations. The research questions for this study were: 1) How do nurses experience their competencies in HHC situations? 2) How were their competencies utilized in HHC according to nurses’ own experience?

## Methods

### Design

A qualitative explorative design was selected for this study. This design was undertaken to achieve better understanding [25] of nurses’ experiences in relation to their competencies in HHC and to discover new areas for research [26].

### Setting and participants

The study was conducted at three municipalities in Western Sweden. Letters of information were sent to clinical managers (CMs) requesting their nurses’ participation in the study. The CMs informed their unit head managers (UHM) about the study, and they in turn mediated contact with participants. Interested nurses were contacted by the researchers (ML and MP) with a request for their participation. The criterion for participation was 1) willingness to describe their experiences in relation to their competencies in HHC; 2) having at least two years’ professional experience as a nurse in HHC; and 3) having undertaken some form of competence developing activities during the last three years. Of the participants, five were RNs and five were ANs. All were female. The age span was 31-54 years old and they had worked between 9 and 35 years in their profession. Two of the participants had experience of working night shifts in HHC.

### Data collection

After giving written consent to being interviewed the nurses chose the location for the interviews. The majority of the interviews were conducted at the nurses’ workplace, except for one interview that was conducted in that nurse’s home. Before the interviews commenced, the interviewer (ML or MP) created a welcoming atmosphere with small talk to make the nurses feel comfortable with the interview situation [27]. Based on the research questions, the following interview questions were formulated: 1) “Could you please tell me about how you experience your competencies in HHC?”, 2) “Could you please tell me about how you experience that your competencies are being utilized in HHC?” In order to gain a deeper understanding, probing questions were posed, for example “Can you tell me more about this?” or “How did you think about that?” To confirm that the nurses’ descriptions were correctly understood, ongoing clarifications were made during the interviews [25]. The interviews lasted 8 h in total, with an individual range of 37-78 min.

### Data analysis

The digitally recorded interviews were transcribed verbatim and analyzed using qualitative content analysis [28, 29]. Initially, the transcribed interviews were read several times to obtain data familiarization and to facilitate an understanding of the whole. Meaning units were extracted, condensed and labelled with a code in accordance with the aim of the study. In the search for themes, the codes were compared and grouped according to different content aspects based on similarities within and differences between themes. The themes were reviewed, defined and named during the analysis process. Continuing the analysis process, the research team discussed their reflections on the codes and themes. This approach was intended to ensure trustworthiness and to keep the balance between the researchers’ preunderstanding of HHC and their openness to the content [30]. The researchers’ preunderstanding is based on several years of work experience in teaching RNs and/or ANs. Two of the researchers teach either in municipal adult education (ML) or clinically within HHC (MP). The other two researchers (LJ and HA) teach at university in nursing programme and specialist nursing programme (district care, prehospital emergency care and emergency nursing). To underline the authenticity of the analysis, a number of quotations are given below. The quotations are labelled with a code in order to safeguard the participants’ integrity. Following this is a presentation of the results.

### Ethics

This study conforms to the ethical principles for research outlined in the Declaration of Helsinki [31] and adheres to Swedish laws and regulations concerning informed consent and confidentiality [32, 33]. All participants received both oral and written information about the study before they gave their written consent to participate.

## Results

The interviews resulted in three themes: Being a capable nurse, Being a useful nurse and Being a subordinate and dependent nurse. The themes are presented in the text below, with illustrative quotations from the interviews.

### Being a capable nurse

Nurses wanted to be capable of taking care of patients, to develop their competencies and to perform their duties in the way required.

Nurses stated that they had a strong desire to do their best for both their patients and the colleagues they worked with. Being a capable nurse involved maintaining control over the situation and feeling confident when carrying out the work. Nurses considered that this was particularly important since patients’ care needs are increasing and more advanced care and treatment are undertaken in HHC situations. Nurses also described the feeling of being capable as essential since it influenced their sense of wellbeing:

*"You feel some kind of satisfaction in your body ... a harmonious feeling that makes you want to continue to work… to advance a little more, to reflect and think in new ways "* (3)

Recognition was also an essential factor in creating the feeling of being a capable nurse. Nurses described the importance of managers’ and colleagues’ recognition of their competencies. Recognition created a feeling of being carefully chosen and strengthened their confidence, generating meaning and pleasure for them in their work. Recognition also meant an opportunity for new work-related responsibilities and invitations to participate in work improvement:

*"The manager is observant in order to discover the competencies available…choosing the right people for new assignments in the team…having my ideas and advice listened to and getting the opportunity to teach colleagues makes me feel pleased"* (5)

However, there were conditions that influenced nurses’ capacity to be capable as nurses. Nurses described their frustration when their competencies were not always utilized, calling that a waste of competencies. An example of waste according to these nurses was the administrative work they had to do, which they described as time-consuming and stealing time from actual nursing care. Competence development activities for nurses were perceived as necessary and obvious, especially when their duties changed. However, sometimes competence development activities were also perceived as a waste of time, when activities did not lead to any work improvement:

*"This training was of no use ... it was a lot of money and education that just vanished into thin air ..." (1)*

### Being a useful nurse

Nurses wanted their work to be useful and to provide good and safe HHC. Being a useful nurse was considered to involve sharing competencies with and learning from other colleagues. Sharing and learning influenced their enthusiasm for work and increased their job satisfaction.

*"... the pleasure of teaching someone else, knowing that I am the one who has taught my colleague something ... makes me feel knowledgeable and needed"* (6)

According to the nurses interviewed, competencies are changeable. They felt responsibility for the transfer of competencies between themselves and their colleagues. This responsibility was considered particularly important since their work was generally carried out in environments with limited opportunities to consult other colleagues. Several nurses emphasized the importance of co-operating and sharing competencies with each other since this influenced their capability to provide quality in care and treatment. This was also essential as a way to reduce the feelings of aloneness and vulnerability resulting from their work situation.

*"There are more tasks to carry out and a lot of colleagues that you have to support…feeling worried if I can do it all... we meet every morning to discuss problems, here I can consult others in the team"* (7)

Nurses described sharing competencies as an obligation. However, this was not always based on willingness. Sharing competencies was essential when nurses felt that colleagues had insufficient competencies, making it impossible to fulfil the requirement to provide good and safe HHC. In contrast, limited opportunity to share competencies created uncertainty and frustration among nurses.

*"I’d find it very disappointing if I couldn’t share my competence with others... if I didn’t have any opportunity to teach others when I have the capability to contribute something"* (9)

Simultaneously, nurses were concerned about their competencies and how they were utilized in their work. Some nurses, considered that there was territorial thinking directed towards colleagues. This affected the sharing of competencies with each other since the attitude existed that no one should believe that anyone was better than anyone else.

*"[after having participated in competence development] it felt clear that it was a while since my colleagues did their studying ... felt the risk of conflict…you should not stand out too much [and] demonstrate that you are better than others..."* (8)

### Being a subordinate and dependent nurse

Nurses interviewed wanted to improve the care given in HHC by utilizing their competencies. Simultaneously, they were subordinate and dependent in relation to their manager and also dependent on the manager’s interest in encouraging nurses’ competence development.

*"…of course I mention to my manager that I want competence development ... time and again [the manager says that] there are no colleagues who can replace me…or it costs too much money…the question is how interested they really are ..."* (3)

Nurses described workplace meetings as important fora for discussion and the exchange of competencies between colleagues. However, this method for learning and competence development was time-consuming and not always perceived as a priority by managers. According to several nurses, managers were an important influence when it came to organizing opportunities for nurses to use and develop their competencies in HHC. Nurses pointed out how important it was for managers to know exactly what competencies they had. However, managers were not always ready to take advantage of nurses’ competencies. Some nurses stated that they had more competencies than managers asked for. There was dissatisfaction among nurses because they were given only limited opportunities to initiate participate in and evaluate development projects in HHC.

*"I felt that I had to take a step back ... I felt I shouldn’t present too many ideas at the same time... [they were] not ready for it"* (8)

There were limitations when it came to nurses’ competence development. According to several nurses, there was inadequate planning and follow-up of their competence development activities. Managers were also uninterested in feedback after completed competence development activities. Nurses felt that it was solely up to them to take the initiative to participate in competence development activities.

*"When participating in competence development activities, our competencies must be used otherwise they will fade away very rapidly... it takes time, willingness and commitment [by managers] to take advantage of what has been learnt... I feel many times that my competencies are not being used afterwards..."* (10)

Lack of appreciation and support from managers generated feelings such as boredom, anger, despair and fatigue with the result that nurses tended to withdraw or give up their ambitions to develop their competencies. There was also criticism towards managers who provided different conditions for competence development depending on whether nurses were working day or night shifts. Nurses who worked at nighttime felt they were forgotten when it came to opportunities for competence development.

*"...when offering competence development activities, the manager hands over notification forms to those interested ... this does not happen to those who work the night shift"* (6)

## Discussion

The results show that nurses understood their expertise in HHC as including knowledge and skills to take care of patients, the ability to develop their competencies, and the capacity to perform their duties in the way required. Other results were nurses’ understanding of their working role as being useful and providing good and safe HHC. To be useful, nurses required conditions allowing them to share their competencies and to learn from their colleagues. The results also showed that the professional development required of nurses was dependent on their unit manager and the manager’s interest in encouraging competence development.

Capra [34] argues that knowledge consists of fundamental principles, rules and laws and can thus resemble a building. This study is about how nurses ‘experienced their expertise and its utilization in HHC situations. Organizationally, nurses found themselves close to their patients and able to meet current requirements and provide nursing care for patients and their relatives. They were also able to guide and give advice to each other and managers. Administration and management must however make efforts to provide conditions allowing the people working in their organization to become skilled nurses. This involves managers understanding what the needs are and what competence each individual and work group must develop based on HHC needs. In instances when managers are not aware of individual nurses’ or team needs this will reflect on HHC performance. Management is an important function and managers must be observant towards the nurses in their team so that they in turn will be willing to be involved and responsible. Each person is unique and affects the way the team works [35].

Managers must show interest and encourage their nurses to undergo competence development that can be guaranteed from ecological, social and economic perspectives. HHC is an arena where patients’ care needs are extensive and complex. Seen from the perspective of economy and developmental changes in society, the character of nurses’ work must change accordingly and thus nurses need to be better prepared. Among other things, this will require of nurses that they are able to understand and practice certain techniques in patient care [36]**.** Munck et al. state [3] for example that the requirements for nurses are high. The increasing use of medical equipment that has increased in advanced home healthcare may be seen as a solution to decreased resources in healthcare. This will affect planning in the long term, making it essential to secure sustainable progress in HHC nurses’ competence development [3, 37]**.** If these factors are not taken into account, they will negatively influence patient safety, together with the shortage of nurses and loss of medical competence. The social perspective involves the perception that nurses’ work is characterized partly by its isolated working conditions and the limited opportunities given for consulting colleagues. Lack of continuity and support in the management of medical equipment may cause feelings of uncertainty and vulnerability in nurses. Cost-effective healthcare to meet the care needs of patients with long-term illnesses requires nurses to have opportunities to undergo regular professional competence development [4]**.** The ecological perspective involves focusing on the environment in which nurses generally work. Lack of expertise may lead to anxiety, uncertainty and a sense of not being able to deal with certain situations. This in turn may render the introduction of new technologies unfeasible [21, 38]. It is vital for nurses to enjoy wellbeing as they do when they feel in control of the situation. Tasks that are considered severe may lead to feelings of inadequacy, as Ellström et al. concluded [38]**.**

Two other studies [36, 39] have shown that nurses’ highest priority was their patients and that they wanted above all to fulfil their patients’ care needs, but that an excessive workload and insufficient influence over their work made them feel inadequate and frustrated. Having sufficient influence over their work and a low level of organizational restriction increased their job satisfaction. A working environment that allowed and encouraged learning and competence development meant a lot for employees’ health, wellbeing and personal development. These factors combined led to qualitative development in the organization. Similarly, studies have shown that the higher the competencies in caregivers, the smaller the risk of healthcare damage to patients and deficiencies in patient safety [40]**.**

Nurses in this study felt that they were in control and provided good and safe care. Providing good and safe care constitutes a growing challenge since more and more patients in HHC are severely ill and in need of advanced medical care. The combination of the needs of patients, colleagues and managers may lead to moral stress for HHC nurses. Reinforcing their skills and specifically their ethical competency may be a way to prevent or reduce moral stress, as Kälvemark-Sporrong et al. declared [41]. Ethical competence can be described as a psychological skill, or “tacit knowledge”. In instances where unit managers supported best practices, nurses also experienced them as ethical practices [42]. Above all, this is important since nurses are more likely to leave their jobs because of moral stress combined with lack of managerial support when trying to act ethically [43]**.** Several studies have shown that psychological stress affects nurses’ ability to listen and respond to patients [44, 45] and that nurses are under great pressure in home healthcare [46, 47]. Nurses may perhaps be able to minimize their stress and powerlessness, given managerial support and affirmation. Ågren-Bolmsjö, Edberg & Sandman [48] stated that difficulties that are more of moral character require discussion in the working group.

In this study, nurses saw themselves as responsible for the transfer of competencies between themselves and their colleagues. The need for competency transfer is especially great since nurses generally work alone in their patients’ homes. This environment offers only limited opportunities to consult colleagues. Collaboration and mutual knowledge transfer is thus essential. This approach allows nurses to provide good quality care and treatment. It is also a way to reduce the feeling of being exposed and vulnerable. One way to meet this demand can be through dialogue in their team. This can be a way to contribute to learning and competence development [35]. This development is based on learning and the sharing of knowledge, leading to an open work environment and the development of better care relationships with patients [49]**.** Many organizations focus on tasks and routine procedures, and avoid discussing interpersonal relationships [50]**.** Where there is little or no time for joint consideration, many development opportunities in work situations may be lost [51]. Conversely, if colleagues have regular team meetings combining team members with different competencies, this often results in improved communication. Routines can be developed if a team has a mix of competencies, and if it promotes frequent and clear internal communication [52]**.** Team members can also help each other to take advantage of new knowledge and understand their own competencies better [53, 54]. This approach has the potential for sustainable development in nursing, attitudes, opinions and discussions. It can thus lead to continued development through creative consideration thus enhancing valuable competencies [55]. Teamwork breaks down boundaries and contributes to the continuity of home healthcare. The result is a highly competent team, which in turn is a prerequisite for good and safe care [18]**.**

What will happen if nothing is done to meet the needs of future HHC? According to this study, three aspects of how HHC nurses experience their competencies in HHC are especially relevant to this question: Being a capable nurse, Being a useful nurse and Being a subordinate and dependent nurse. These aspects may be perceived as interacting with each other and forming a whole [34]**.** According to the Swedish National Board of Health and Welfare [4]**,** it is important to bridge the gap between the competencies demanded by HHC and the competencies possessed by nurses. There are unfortunately forces acting against nurses’ competence development. What will happen if nurses are not allowed to use and develop their competencies as mentioned earlier is that the economic, social and environmental perspectives will be affected. Meeting patients’ HHC needs in the future will require considerable investment in competence development. Managers who support organizational conditions that provide more opportunities for nurses’ competence development programs will be making an investment in the future [56]**.**

## Strengths and limitations

There are factors that influence research such as the researcher’s background, position and perspectives [57]. To reduce conceivable biases, this study was based on the researchers’ open attitude, receptivity and sensitivity to the aim of the study. The main strength of this study has been that the nurses interviewed came from different workplaces and municipalities. They also had significant experience of working as nurses. However, only a small number of nurses agreed to participate in the study, among them no males. This means that nurses’ experiences of their competencies in HHC might lack a degree of variation. Another limitation is related to the method of data analysis. This study used a qualitative content analysis to describe variations by identifying similarities and differences in the textual content as whole rather than specifically examining groups. Therefore, this study cannot say anything about similarities and differences between RNs and ANs in how they experienced their competencies. The findings from this study cannot be generalized but they may be transferred to similar contexts despite these limitations.

## Conclusion

Nurses’ competencies in HHC constitute a complex issue. This study highlights the desire of HHC nurses to be capable of caring for their patients and also to develop their competencies. Nurses want their work to be useful. They want provide good and safe HHC and to improve care by fully utilizing their competencies.

HHC nurses provide advanced care and treatment in patients’ homes. To ensure that patients are given good and safe HHC, nurses need comprehensive competencies, requiring continuous learning of both theoretical knowledge and practical skills. This is particularly important since the number of patients with serious illnesses needing advanced medical treatment is increasing in HHC.

Managers can and should be significantly influential in recognizing nurses’ competencies and in encouraging their further development. It is essential for managers to shoulder this responsibility and to give nurses sufficient recognition and strong support in their competence development. Otherwise, working in unstructured and perhaps even chaotic environments in which nurses do not feel that they have adequate control can increase the risk for burnout and staff turnover.

Further research is needed to gain deeper understanding of nurses’ competencies and how these are utilized. It is important for such research also to include managers’, patients’ and relatives’ perspectives.

## Abbreviations

AN: Assistant nurse
CM: Clinical manager
HHC: Home healthcare
RN: Registered nurse
UHM: Unit head manager
